# Special Issue “Advancements in Cancer Biomarkers”

**DOI:** 10.3390/ijms27156580

**Published:** 2026-07-24

**Authors:** Michael Hannides, Christos Mikropoulos

**Affiliations:** Clinical Oncology, Royal Surrey NHS Foundation Trust, Egerton Road, Surrey, Guildford GU2 7XX, UK; christos.mikropoulos@nhs.net

## 1. Introduction

This Special Issue, “Advancements in Cancer Biomarkers”, focuses on recent developments in cancer biomarker research, providing an updated perspective on the rapidly evolving field of molecular oncology. Cancer biomarkers have become indispensable in modern oncology, contributing to early detection, diagnosis, prognostic assessment, therapeutic decision-making, and treatment monitoring. Continuous advances in molecular biology, high-throughput technologies, and computational analyses have accelerated the discovery and clinical translation of novel biomarkers, paving the way for more precise and personalised cancer management.

With the aim of bringing together experts from diverse disciplines, this Special Issue presents original research articles and comprehensive reviews that showcase recent advances in cancer biomarker discovery, validation, and clinical application across a broad spectrum of malignancies. The published studies span foundational, translational, and clinical research, reflecting the multidisciplinary nature of contemporary cancer biomarker research and highlighting emerging approaches with the potential to improve patient outcomes.

Overall, this Special Issue comprises 14 peer-reviewed articles contributed by researchers from nine countries across four continents, illustrating the global interest and collaborative efforts in advancing cancer biomarker research. Since its publication, the collection has attracted more than 39,000 views, underscoring its relevance to the scientific community and the growing demand for comprehensive insights into this rapidly expanding field.

## 2. Advancements

A central theme emerging from this issue is the growing impact of integrative multi-omics analysis in the discovery of novel biomarkers and the illumination of complex molecular mechanisms underlying tumour development. By combining genomic, transcriptomic, epigenomic, and bioinformatic analyses, these studies demonstrate how comprehensive molecular profiling can identify clinically relevant biomarkers and potential therapeutic targets.

In hepatocellular carcinoma (HCC), NUP205, a component of the nuclear pore complex, is identified as a promising prognostic biomarker associated with disease progression, drug responsiveness and patient outcomes [contribution 1]. Utilising integrated computational and bioinformatics analyses, the cell-cycle regulator CDK1 acts as both an immunomodulatory regulator and a potential prognostic biomarker in liver fibrosis-associated HCC [contribution 2], highlighting the interplay between cell-cycle dysregulation, the tumour immune microenvironment, and hepatocarcinogenesis.

Integrative multi-omics analyses also provide new insights into the epigenetic mechanisms driving cancer pathogenesis. In colorectal cancer, TNXB is the most significantly epigenetically dysregulated gene in a filtered epigenome-wide analysis, demonstrating its association with poor overall survival and its potential as a prognostic biomarker [contribution 3]. Similarly, in non-small cell lung cancer (NSCLC), the transcription factors SOX18 and SOX30 play distinct, divergent roles in tumour development and progression, and these findings uncover a previously unrecognised regulatory axis that may serve as both a biomarker of disease biology and a promising therapeutic target [contribution 4].

Collectively, these studies [contributions 1–4] illustrate the power of integrative multi-omics strategies to uncover molecular determinants of cancer progression, identify biomarkers with prognostic significance, and reveal novel pathways that may be exploited for precision oncology.

Another prominent focus of this Special Issue is the development of minimally invasive biomarkers, reflecting the increasing shift toward liquid biopsy-based approaches for cancer diagnosis, prognosis, and disease monitoring. By analysing circulating tumour-derived components and other accessible biological specimens, these studies highlight the potential of non-invasive biomarkers to improve clinical decision-making while reducing the need for invasive tissue sampling.

In lung cancer, tumour hybrid cells (THCs) and soluble immune checkpoints (sICs) can serve as biomarkers for stratifying metastatic progression and patient survival [contribution 5]. Among the circulating immune markers evaluated, soluble CTLA-4, 4-1BB, LAG-3, and TIM-3 demonstrate the greatest discriminatory capacity for disease progression and clinical outcomes [contribution 5], underscoring the prognostic value of immune-related circulating biomarkers.

Expanding the concept of liquid biopsy beyond blood-based analyses, extracellular vesicles (EVs) isolated from gastric juices can function as biomarkers for gastric cancer. Through cryo-electron microscopy, there are significant morphological differences between patients with and without gastric cancer [contribution 6]. In particular, increased EV size and the CD9-positive-to-CD9-negative vesicle ratio distinguish patients with gastric cancer from healthy controls, highlighting gastric juice-derived EVs as a promising tissue-specific and minimally invasive diagnostic platform.

Furthermore, circulating microRNAs serve as preoperative biomarkers to differentiate benign from malignant thyroid nodules. Specifically, miR-221 is a highly accurate diagnostic biomarker, achieving 100% sensitivity and specificity within the studied patient cohort [contribution 7]. Although validation in larger populations is warranted, these findings support the growing clinical potential of circulating microRNAs to improve the management of indeterminate thyroid nodules and reduce unnecessary surgical interventions.

These published studies [contributions 5–7] demonstrate the rapid evolution of minimally invasive biomarker technologies and their potential to enhance early cancer detection, risk stratification, and personalised patient management across different malignancies.

The tumour microenvironment emerges as a key component of research, and new research illustrates how interactions between tumour cells and their surrounding stromal, immune, and extracellular components shape cancer behaviour and influence clinical outcomes.

Using three-dimensional spheroid models, four distinct subtypes of cancer-associated fibroblasts (CAFs) derived from breast cancer cultures were developed and comprehensively characterised [contribution 8]. This revealed marked functional heterogeneity within the CAF population, with specific subtypes exhibiting increased resistance to doxorubicin, cisplatin, and tamoxifen, while others acquire enhanced metastatic potential under hypoxic conditions [contribution 8]. These observations reinforce the importance of stromal heterogeneity as both a determinant of therapeutic response and a potential source of novel biomarkers and treatment targets.

Complementing these findings, transcriptomic analyses and zebrafish xenograft models indicate that the platelet-derived molecule Defensin Alpha 1/3 (DEFA1/3) promotes tumour cell proliferation and migration in pancreatic ductal adenocarcinoma by inducing the expression of genes associated with aggressive disease progression [contribution 9]. Furthermore, analysis of The Cancer Genome Atlas data links elevated DEFA1/3 expression with poorer patient survival and increased immune cell infiltration, highlighting its potential as both a prognostic biomarker and a therapeutic target.

Building on these observations, a comprehensive review examines the multifaceted role of transforming growth factor-β (TGF-β) signalling in lung, colorectal, and breast cancers. Dysregulated TGF-β signalling contributes to tumour progression by promoting immune evasion, epithelial–mesenchymal transition, metastasis, and resistance to therapy [contribution 10], with the potential for emerging therapeutic strategies to target this pathway.

Taken together, these contributions [contributions 8–10] highlight the tumour microenvironment as a dynamic and clinically relevant source of biomarkers, providing important insights into the molecular mechanisms driving cancer progression and therapeutic resistance while identifying new opportunities for precision-targeted interventions.

Biomarkers that inform therapeutic response and guide precision oncology are also recognised, and research in this Special Issue represents both the progress made in identifying predictive biomarkers for treatment response and the ongoing need for clinically validated markers to optimise therapeutic selection across various malignancies.

In non-small cell lung cancer (NSCLC), the antimicrobial agent nitroxoline enhances radiosensitivity in vitro by suppressing the STAT3–AKT–mTOR signalling pathway [contribution 11]. The findings suggest that modulation of this pathway may improve radiotherapy efficacy while identifying molecular signatures associated with treatment response. Expanding on biomarker-guided radiotherapy, a comprehensive narrative review of biomarkers for patients undergoing stereotactic ablative radiotherapy (SABR) across multiple tumour types discusses a broad spectrum of emerging biomarkers [contribution 12]. Biomarkers such as circulating tumour DNA, extracellular vesicles, immune-based biomarkers, liquid biopsy approaches, and radiomic signatures highlight their potential to refine patient selection, predict therapeutic outcomes, and support increasingly personalised radiotherapy strategies.

Nevertheless, several contributions emphasise the challenges that remain before precision oncology can be fully realised. There are still a lack of clinically validated genomic biomarkers to guide treatment selection in breast cancer, despite rapid advances in molecular profiling technologies [contribution 13]. Similarly, in multiple myeloma, there is a call for emerging molecular targets for chimeric antigen receptor (CAR)-based immunotherapies, underscoring the need to expand beyond current target antigens to improve treatment efficacy, overcome resistance, and achieve more durable clinical responses [contribution 14].

## 3. Summary

The contributions presented in this Special Issue highlight several overarching trends shaping the future of cancer biomarker research. First, there is a clear transition toward integrative, multidimensional approaches that combine genomic, epigenomic, transcriptomic, proteomic, and computational analyses to capture the complexity of cancer biology. Second, increasing recognition of the tumour microenvironment and systemic host factors has revealed their critical roles in regulating disease progression, immune interactions, and therapeutic response. Third, the growing development of minimally invasive biomarkers, including circulating tumour cells, extracellular vesicles, and microRNAs, reflects the movement toward dynamic, real-time monitoring of disease evolution and treatment efficacy.

## 4. Obstacles

Despite these important advances, several challenges remain. Many promising candidate biomarkers remain in early stages of development and require validation in large, independent, and diverse patient cohorts. Furthermore, challenges related to analytical standardisation, reproducibility, regulatory approval, and integration into routine clinical workflows continue to limit widespread implementation. In particular, translating complex multi-omics signatures into robust, accessible, and clinically actionable tools remains a major challenge. Addressing these barriers will require continued interdisciplinary collaboration between scientists, clinicians, bioinformaticians, and industry partners to ensure that emerging biomarker discoveries ultimately improve patient care.

## 5. Conclusions

In conclusion, this Special Issue provides a comprehensive overview of recent advancements in cancer biomarker research, encompassing fundamental discoveries, technological innovations, and emerging translational applications. A summary of biomarkers, key findings and possible implications is listed in Table 1. The editors sincerely thank all contributing authors for sharing their valuable expertise, research, and insights, and the reviewers for their constructive evaluations, which have enhanced the quality of this collection. We hope that the findings and perspectives presented in this Special Issue will inspire further interdisciplinary research, accelerate biomarker discovery and validation, and ultimately support the development of improved diagnostic, prognostic, and therapeutic strategies for patients with cancer.

## Figures and Tables

**Table 1:** Summary of papers including biomarkers, key findings and potential implications.

Study	Biomarker	Tumour Type	Findings	Implications
Jeong, Lee and Kim.	NUP205 (nucleoporin)	Liver hepatocellular carcinoma	Elevated NUP205 expression is associated with advanced clinicopathological features, resistance to multiple chemotherapeutic agents and poor prognosis. Upregulation of NUP205 is driven by epigenetic alterations and miRNA interactions, which also influence drug sensitivity.	NUP205 is a candidate prognostic biomarker. NUP205 may influence drug responsiveness across multiple therapeutic classes. NUP205 knockdown improved sensitivity to doxorubicin.
Gong et al.	STAT3 (transcription factor)	Non-small cell lung cancer (NSCLC)	In NSCLC, elevated STAT3 expression and phosphorylation are associated with advanced disease stages, poor overall survival, and reduced response to targeted therapy. The antimicrobial nitroxoline enhanced radiation-induced cytotoxicity by suppressing the STAT3-AKT-mTOR signalling pathway.	Potential of repurposing nitroxoline as a radiosensitiser to improve radiotherapy outcomes in patients with NSCLC.
Sáenz de Santa María-Diez et al.	Tumour hybrid cells (THCs) and soluble immune checkpoints (sICs)	Lung cancer	The number of THCs and levels of sCTLA-4, s-41BB, sLAG-3, and sTIM-3 (sICs) exhibited the strongest discrimination for metastasis. THCs, sLAG-3, and sTIM-3 also distinguished between surviving and non-surviving patients. Soluble PD-L1 (sPD-L1) levels were significantly higher in patients with metastatic disease and in non-survivors.	LAG-3 may serve as a circulating marker of advanced immune dysfunction. The integration of THCs and sICs could support personalised medicine by identifying patients at higher risk of early metastasis or poor survival who may benefit from intensified surveillance, combination immunotherapies, or early systemic intervention. The heterogeneous clinical response to PD-1/PD-L1 blockade highlights the limitations of a single immune checkpoint as a prognostic or predictive marker, reinforcing the value of multiplex immune profiling.
Olbromski et al.	SOX (transcription factors)	Non-small-cell lung cancer (NSCLC)	SOX18 and SOX30 play divergent roles in NSCLC progression. SOX18 functions as a pro-oncogenic factor driving angiogenesis and tumour–stroma interactions. SOX30 acts as an epigenetically silenced tumour suppressor.	Regulation of SOX18 by miR-24-3p highlights a potential therapeutic vulnerability that could be targeted through epigenetic or miRNA-based interventions.
Gonzalez-Ruiz et al.	Platelet-associated Defensin Alpha 1/3 (DEFA1/3)	Pancreatic ductal adenocarcinoma (PDAC)	Elevated platelets in PDAC are associated with poor prognosis, reduced treatment efficacy, and enhanced metastatic potential. Defensin-rich platelets promote proliferation, migration, and spheroid growth of pancreatic cancer cells and upregulate aggressiveness-related genes, leading to adverse clinical outcomes.	Platelet-derived *DEFA1/3* act as active contributors to the malignant pancreatic ductal cell adenocarcinoma phenotype and may serve as biomarkers or therapeutic targets.
Ilyina et al.	Cancer-associated fibroblasts (CAFs)	Breast adenocarcinoma	CAF cell subtypes CAF-S4 and CAF-S1 were sensitive to the action of doxorubicin, cisplatin and tamoxifen. CAF-S2 cells exhibited the highest level of resistance to the antitumour agents. Hypoxia-activated CAF-S4 has been shown to stimulate the metastatic potential of triple-negative breast cancer cells in a heterotypic spheroid model.	A starting point for developing novel therapeutic strategies targeting CAFs and their interactions with cancer cells.
Matei et al.	Circulating microRNAs (miRNAs)	Thyroid carcinoma	Strong potential diagnostic utility for miR-221, and to a lesser extent for miR-146a, in distinguishing malignant from benign thyroid nodules in the preoperative setting.	Circulating miR-221 may serve as a highly accurate, non-invasive biomarker for thyroid malignancy. There is potential clinical utility in the pre-surgical setting, especially in cases with indeterminate cytology.
Pilo et al.	Tumour suppressor gene Tenascin XB (TNXB)	Colorectal cancer	TNXB was the most epigenetically dysregulated gene, found to be hypomethylated and downregulated in colorectal cancer. This leads to increased cell migration and proliferation in colorectal cell lines, suggesting a role for this gene as a tumour suppressor. TNXB was epigenetically regulated in a dose-dependent manner.	Provides a novel tumour-suppressor gene which could serve as an indicator of malignant potential and progression in colorectal cancer
Skryabin et al.	Gastric juice extracellular vesicles (EVs)	Gastric adenocarcinoma	Vesicles obtained from the gastric juices of cancer patients had a markedly larger mean diameter. The ratio of CD9-positive to CD9-negative EV samples differed between cancer patients and healthy donors.	The increased presence of CD9-positive extracellular vesicles in the gastric juice of cancer patients may have significant diagnostic and prognostic implications as a minimally invasive liquid biomarker.
Qin and Li.	Cyclin-dependent kinase 1 (CKD 1)	Liver fibrosis-associated hepatocellular carcinoma	CDK1 was significantly overexpressed in HCC tissues compared with normal controls. Elevated CDK1 expression correlated with reduced overall survival and advanced tumour staging. Strong associations were found between CDK1 levels and immunosuppressive cell infiltration, particularly regulatory T cells and myeloid-derived suppressor cells. Simulations revealed high-affinity binding of CDK1 to kinase inhibitors, with alvocidib exhibiting optimal binding stability relative to seliciclib and alsterpaullone.	CDK1 serves as both a prognostic biomarker and an immunomodulatory regulatory therapeutic target. The expression level of CDK1 can serve as a basis for personalised treatment in patients with liver fibrosis. The complexity of CDK1 in cell cycle regulation limits the use of a single CDK1 inhibitor.
Metawe et al. (Review)	Circulating tumour DNA (ctDNA)	Renal cell carcinoma	Longitudinal monitoring of ctDNA has demonstrated potential as a prognostic biomarker.	Can identify individuals at higher risk of early relapse following SABR.
		NSCLC	The prognostic value of ctDNA prior to radiotherapy was demonstrated in patients with undetectable ctDNA, who experienced significantly improved progression-free and overall survival.	ctDNA can serve as a non-invasive biomarker to distinguish between those more likely to benefit from stereotactic ablative radiotherapy (SABR).
	Extracellular vesicles (EVs)	Prostate cancer	Stratification of patients by baseline prostate-specific membrane antigen-positive (PSMA+) EV levels revealed that lower concentrations were significantly associated with superior post-treatment outcomes. Those with low baseline PSMA+ EV levels had longer biochemical progression-free survival after SABR, but no benefit was observed in patients with high baseline levels.	PSMA+ EVs may help identify individuals most likely to benefit from metastasis-directed therapy.
	Immune biomarkers	NSCLC and melanoma	Responders (post-SABR fractions) demonstrated a decline in cfDNA (cell-free DNA) from the second to third treatment fractions with an increase in CD8^+^PD-L1^+^ T cells, while non-responders showed elevated CD8^+^PD-1^+^ T cells. Immune profiling of patients with metastatic melanoma treated with pembrolizumab showed that the proliferation marker Ki-67 was significantly upregulated in PD-1^+^ CD8^+^ T cells three weeks after therapy initiation.	Immune biomarkers offer promise as early predictors of treatment response. Potential as a pharmacodynamic biomarker in checkpoint blockade therapy.
Cecerska-Heryć et al. (Review)	Transforming growth factor-β (TGF-β)	Lung cancer	Elevated levels of TGF-β in extracellular vesicles are correlated with resistance to immune checkpoint inhibitors, shorter progression-free survival, and poorer overall survival. Lucanix, a non-viral, gene-based tumour cell vaccine targeting TGF-β2, was examined in a Phase III trial for NSCLC; however, the study’s survival target was not met, indicating no significant difference	TGF-β serves as a prognostic and predictive biomarker in lung cancer.
		Breast cancer	TGF-β signalling significantly contributes to breast cancer progression by modulating epithelial–mesenchymal transition, immune escape, and chemoresistance. 264RAD, a human monoclonal antibody targeting the αvβ6 integrin, can inhibit tumour development and metastasis in vivo.	Targeting the TGF-β pathway, either directly or in combination with immunotherapy, represents a promising strategy to overcome treatment resistance and improve outcomes. Therapeutic targeting of the TGF-β pathway, particularly through inhibitors of integrins or downstream effectors like SOX4, offers promising potential for improving outcomes in aggressive breast cancer subtypes such as triple-negative breast cancer. Patients with high-risk and trastuzumab-resistant breast cancer may benefit from targeting αvβ6 with 264RAD antibody alone or in conjunction with trastuzumab.
Beecher et al. (Review)	Genomic biomarkers BRCA1/2	Breast cancer	Poly (ADP-ribose) polymerase (PARP) inhibitor (PARPi) treatment has shown benefit in patients with a homologous recombination deficiency phenotype.	In a phase III trial, second-line olaparib treatment led to significantly longer disease-free survival and overall survival in early high-risk HER2- breast cancer patients with confirmed BRCA1 or BRCA germline mutations.
	PIK3CA		The current biomarkers predictive of PARPi sensitivity are germline BRCA1/2 (gBRAC1/2) and PALB2 mutation. The PI3K/AKT/mTOR pathway can act as an oncogenic driver of proliferation in cancer. PI3K catalytic subunit (PIK3CA) mutations are detected in approximately 30% of breast cancers and are notably more common in ER+ and HER2+ disease. PIK3CA mutations predict resistance to a range of chemotherapies.	In a phase III trial, inavolisib (plus palbociclib and fulvestrant) led to a significant overall survival benefit in patients with PIK3CA-mutated, HR+ HER2- advanced breast cancers. In the neoadjuvant setting, HER2+ breast cancer showed PIK3CA mutations were associated with poorer outcomes in all treatment groups. The status of PIK3CA is crucial for personalised therapeutic strategies in breast cancer.
	ESR1		In HR+ breast cancer, resistance often emerges over time with mutation of the ESR1 ligand-binding domain that encodes the oestrogen receptor alpha.	Elacestrant has demonstrated significant efficacy, with extended progression-free survival, amongst patients with ESR1 mutations. Lasofoxifene, a novel selective ER modulator, has also been shown to improve the clinical benefit rate in patients with ESR1 mutations who progressed on an aromatase inhibitor plus a CDK4/6 inhibitor. Monitoring the emergence and persistence of ESR1 mutations in blood samples offers a promising non-invasive approach for real-time disease recurrence monitoring and guidance on therapeutic strategies.
	HER2/ERBB		Amplification of the ERBB2 gene intrinsically defines the HER2+ class of breast cancer.	While ERBB2 gene amplification is a long-established predictive biomarker of response, the mutation status of the gene and its binding partner, HER3/ERBB3, is now emerging as a potential predictive biomarker.
	CCNE1		CCNE1 amplification may be relevant as a genomic marker of resistance to CDK4/6 inhibitors. High CNE1 mRNA expression has been associated with resistance to Palbociclib and a poorer prognosis in some studies.	Potential role in guiding therapeutic decisions rather than serving solely as a prognostic indicator.
Vorontsova et al. (Review)	G Protein-Coupled Receptor Class C Group 5 Member D (GPRC5D)	Multiple myeloma	High expression stability in myeloma cells. Clinical efficacy was confirmed in patients with relapsing or refractory myeloma who had previously received BCMA-targeted therapy	GPRC5D is a promising target for cellular therapy in resistant or relapsing myeloma. The ongoing development of GPRC5D-targeted bispecific and allogeneic platforms opens new opportunities for treatment.
	B-Cell Maturation Antigen (BCMA)		*BCMA* is expressed with high density on the surface of malignant plasma cells. High expression level correlates with the intensity of the anti-tumour response, the likelihood of achieving complete remission, and the duration of relapse-free and overall survival. antigen-negative status is linked to the presence of soluble BCMA (sBCMA), which circulates in the blood and competes with membrane-bound BCMA for the CAR, thereby reducing therapeutic efficacy.	The long-term efficacy of BCMA-specific CAR T therapy remains limited. Combined therapeutic strategies that stipulate the co-administration of BCMA-targeted CAR T cells with immune checkpoint inhibitors can decrease the level of soluble BCMA (sBCMA) and increase the expression of membrane BCMA (mBCMA) on the surface of tumour cells, thereby boosting the efficacy of CAR T therapy.

